# Cancer testis antigens expression in mesothelioma: role of DNA methylation and bioimmunotherapeutic implications

**DOI:** 10.1038/sj.bjc.6600174

**Published:** 2002-03-18

**Authors:** L Sigalotti, S Coral, M Altomonte, L Natali, G Gaudino, P Cacciotti, R Libener, F Colizzi, G Vianale, F Martini, M Tognon, A Jungbluth, J Cebon, E Maraskovsky, L Mutti, M Maio

**Affiliations:** Cancer Bioimmunotherapy Unit, Centro di Riferimento Oncologico, IRCCS, 33081 Aviano, Italy; University of Piemonte Orientale ‘A. Avogadro’ Department of Medical Sciences, 28100 Novara, Italy; Azienda Ospedaliera ‘SS Antonio e Biagio’, 15100 Alessandria, Italy; University ‘G D'Annunzio’, Department of Oncology and Neuroscience, 66100 Chieti, Italy; University of Ferrara, Department of Morphology and Embryology, 44100 Ferrara, Italy; Ludwig Institute for Cancer Research, Memorial Sloan-Kettering Cancer Center, 1275 York Avenue, New York, NY 10021, USA; Ludwig Institute for Cancer Research, Austin and Repatriation Medical Centre, Heidelberg, Victoria 3084, Australia; Health Authority 11 Piemonte, Laboratory of Clinical Oncology and IRCCS ‘S Maugeri’ Foundation, 27100 Pavia, Italy

**Keywords:** mesothelioma, immunotherapy, 5-aza-2′-deoxycytidine, cancer testis antigens, methylation

## Abstract

Recent evidences suggest that malignant mesothelioma may be sensitive to immunotherapy; however, little is known about malignant mesothelioma-associated tumour antigens. Focusing on cancer/testis antigens, the expression of well-characterised immunogenic tumour-associated antigens was investigated in malignant mesothelioma cells. At variance with MAGE-4 and NY-ESO-1, malignant mesothelioma cells frequently expressed MAGE-1, -2 and -3, GAGE 1-2, GAGE 1-6, SSX-2 and SSX 1-5, and distinct malignant mesothelioma cells concomitantly expressed at least four cancer/testis antigens. Additionally, the tumour-associated antigens RAGE-1 was expressed at high levels in both benign and malignant mesothelial cells. Lastly, treatment with the DNA hypomethylating agent 5-aza-2′-deoxycytidine induced and up-regulated the expression of the cancer/testis antigen examined in malignant mesothelioma cells. Overall, these findings strongly suggest that cancer/testis antigens-based immunotherapy may represent a suitable therapeutic approach to malignant mesothelioma, and foresee the clinical use of 5-aza-2′-deoxycytidine to design new chemo-immunotherapeutic strategies in malignant mesothelioma patients.

*British Journal of Cancer* (2002) **86**, 979–982. DOI: 10.1038/sj/bjc/6600174 www.bjcancer.com

© 2002 Cancer Research UK

## 

No conventional therapy prolongs survival of malignant mesothelioma (MM) patients (Lee *et al*, 2000); however, immunotherapy may have a positive impact in the treatment of MM. In fact, MM cells can present recall antigens to the immune system (Mutti *et al*, 1998), and systemic administration of IL-12 generated immune responses to MM in murine models (Caminschi *et al*, 1998). Additionally, tumour-reactive antibodies were detected in sera of MM patients (Robinson *et al*, 1998), and non-specific immunotherapy showed some clinical efficacy in human MM (Castagneto *et al*, 2001). Despite these evidences, little is known about MM-associated tumour antigens that may represent useful therapeutic targets to implement immunotherapeutic approaches in MM patients.

Among known immunogenic tumour-associated antigens (TAA) (for review see Traversari, 1999), cancer testis antigens (CTA) including MAGE, GAGE and SSX gene families, and NY-ESO-1, are expressed in solid tumours of different histotype but not in normal tissues except testis (Traversari, 1999). Due to their unique tissue distribution, and recognition by cytotoxic T lymphocytes (CTL) and/or by B lymphocytes, CTA represent useful therapeutic targets in solid malignancies (Traversari, 1999).

To extend to MM patients CTA-based immunotherapeutic approaches that are proving promising in solid tumours (Marchand *et al*, 1999; Nishiyama *et al*, 2001), we analysed the expression of well-characterised CTA by mesothelial and MM cells.

Furthermore, due to the demonstrated role of DNA methylation in regulating gene expression (Jones and Takai, 2001), and since promoter methylation is involved in the expression of MAGE genes in human melanoma (De Smet *et al*, 1996, 1999; Sigalotti *et al*, 2002), we investigated the role of the DNA hypomethylating agent 5-aza-2′-deoxycytidine (5-AZA-CdR) in regulating the differential distribution of CTA and their constitutive levels of expression in MM.

Our results provide the first evidence that different CTA can be co-expressed in and among MM specimens, and also highlight that DNA methylation accounts for their heterogeneous distribution in individual MM tissues.

## MATERIALS AND METHODS

### Mesothelial and mesothelioma cells and 5-AZA-CdR treatment

Primary and long-term cultures of MM cells from pleural effusion of MM patients, and cultures of mesothelial cells from pleural effusion of patients with heart failure were obtained and grown as previously described (Mutti *et al*, 1998). Primary cultures were utilised between the second and third passage *in vitro*. Treatment with 5-AZA-CdR (Sigma Chemical Co., St. Louis, MO, USA) was performed as described (Coral *et al*, 1999).

### Monoclonal antibodies, antisera, reagents and biochemical assays

The anti-NY-ESO-1 mAb ES121 has been previously described (Jungbluth *et al*, 2001). The anti-NY-ESO-1 rabbit antiserum was obtained from 20-week-old NZW female rabbit immunised at weekly intervals with subcutaneous injections of 1 mg of recombinant NY-ESO-1 protein. Immunoprecipitation, SDS–PAGE, and Western blotting were performed as described (Maio *et al*, 1991).

### Reverse transcription (RT) polymerase chain reaction (PCR) analysis and competitive PCR

Total RNA extraction and RT–PCR reactions were performed as described (Coral *et al*, 1999). Oligonucleotide primer sequences and gene-specific PCR amplification programs utilised have been defined for MAGE-1, -2, -3, -4 (Brasseur *et al*, 1995), NY-ESO-1 (Jäger *et al*, 1998), GAGE 1-2 (Van den Eynde *et al*, 1995), GAGE 1-6 (Van den Eynde *et al*, 1995), SSX 1-5 (dos Santos *et al*, 2000), SSX-2 (Sahin *et al*, 2000), RAGE-1 (Neumann *et al*, 1998), tyrosinase and Melan-A/MART-1 (van Elsas *et al*, 1996). The integrity of each RNA and oligo-dT-synthesised cDNA sample was confirmed by the amplification of the β-actin housekeeping gene (Coral *et al*, 1999). Ten μl of each RT–PCR sample were run on a 2% agarose gel and visualised by ethidium bromide staining.

The level of expression of distinct antigens was scored according to the intensity of the specific RT–PCR product, which was obtained by densitometric analysis of ethidium bromide-stained agarose gels using a Gel Doc 2000 documentation system and the QuantityOne densitometric analysis software (Bio-Rad, Milan, Italy). The intensity of RT–PCR products were compared to that of the reference human melanoma cell line Mel 142 (MAGE-1-, -2-, -3-, -4-, GAGE 1-2-, GAGE 1-6-, SSX-2- and SSX 1-5-positive) or human fibrosarcoma cell line HT1080 (NY-ESO-1-positive) or human renal cell carcinoma cell line LE9211 (RAGE-1-positive). Samples were scored −, no RT–PCR product detectable; +, expression level <10% to that of the appropriate reference cell line; ++, expression level >10% to that of the appropriate reference cell line. Competitive PCR for MAGE-3 and β-actin was performed as described (Sigalotti *et al*, 2002).

## RESULTS

### CTA expression in mesothelial and MM cells

Three primary cultures (MES-CM98, MES-MM98, and MES-OC99) and three long-term cultures (MPP-89, MES-1, and MES-2) of MM cells were analysed for their constitutive expression of MAGE-1, -2, -3, -4, NY-ESO-1, GAGE 1-2, GAGE 1-6, SSX-2, and SSX 1-5. RT–PCR analysis revealed frequent expression of CTA belonging to the MAGE, GAGE and SSX gene families; in contrast, a reduced frequency of MAGE-4, and NY-ESO-1 was found (Table 1

**Table 1:**
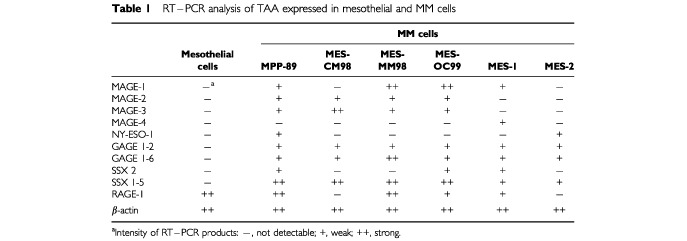
RT–PCR analysis of TAA expressed in mesothelial and MM cells

). None of the CTA examined were detected in mesothelial cells (Table 1).

Noteworthy, four out of six MM cells investigated expressed the TAA RAGE-1 (Table 1), and its expression was also detected in three different primary cultures of mesothelial cells (representative results are reported in Table 1).

Consistent with their classification as melanocyte differentiation antigens, no expression of Melan-A/MART-1 and tyrosinase was detected in mesothelial and MM cells (data not shown).

### Induction and up-regulation of CTA expression by 5-AZA-CdR in MM cells

In order to determine whether DNA methylation could account for the heterogeneous expression of CTA in MM, RT–PCR analysis of CTA expression was performed on total RNA from MPP-89, MES-MM98, MES-OC99, MES-1, and MES-2 MM cells, treated with the DNA hypomethylating agent 5-AZA-CdR and compared with untreated controls. Treatment with 5-AZA-CdR consistently induced the expression of MAGE-1, -2, -3 and -4, NY-ESO-1, and SSX-2 in MM cells constitutively negative for one or more of these CTA (Table 2

**Table 2:**
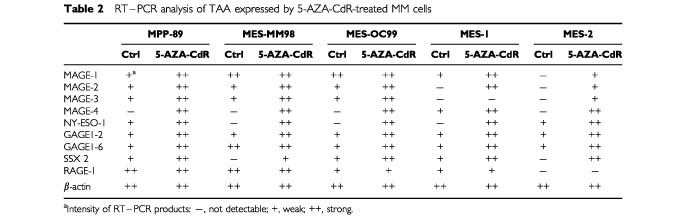
RT–PCR analysis of TAA expressed by 5-AZA-CdR-treated MM cells

, Figure 1

**Figure 1:**
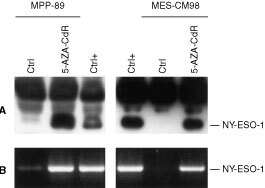
Expression of NY-ESO-1 in 5-AZA-CdR-treated MM cells. (**A**) Cell lysates of MPP-89 and MES-CM98 MM cells treated (5-AZA-CdR) or not (Ctrl) with 5-AZA-CdR for 48 h, and of NY-ESO-1-positive fibrosarcoma cells HT1080 (Ctrl+), were immunoprecipitated by an anti-NY-ESO-1 rabbit antiserum, size-fractionated by a 13% one-dimensional SDS–PAGE under reducing conditions, and blotted onto Hybond-C super transfer nitrocellulose membranes. Then, membranes were incubated with 1 μg ml^−1^ of ES121 anti-NY-ESO-1 mAb, and further processed to be developed by the enhanced chemiluminescence technique. (**B**) Total RNA was extracted from MPP-89 and MES-CM98 MM cells treated (5-AZA-CdR) or not (Ctrl) with 5-AZA-CdR for 48 h, and from NY-ESO-1-positive fibrosarcoma cells HT1080 (Ctrl+), and RT–PCR was performed using NY-ESO-1-specific primer pair. PCR products were size-fractionated on a 2% agarose gel and visualised by ethidium bromide staining.

), with the exception of MAGE-3 in MES-1 MM cells (Table 2). Furthermore, the intensity of RT–PCR products for CTA that were constitutively expressed by MM cells was invariantly higher in 5-AZA-CdR-treated cells (Table 2, Figure 1). Consistently, competitive RT–PCR analysis revealed a six to 10-fold increase in MAGE-3 expression in 5-AZA-CdR-treated MPP-89 and MES-OC99 cells, respectively. As expected, no induction of Melan-A/MART-1 expression was observed following 5-AZA-CdR treatment in MM cells (data not shown).

### Biochemical analysis of NY-ESO-1 expressed by 5-AZA-CdR-treated MM cells

To assess whether the induction/up-regulation of CTA expression observed at mRNA level was followed by the production of the respective protein, immunoprecipitation and Western blotting for NY-ESO-1 were performed on MPP-89 and MES-CM98 MM cells, treated or not with 5-AZA-CdR. 5-AZA-CdR strongly up-regulated the expression of NY-ESO-1 protein in MPP-89 MM cells (Figure 1), and induced *de novo* expression of NY-ESO-1 protein in MES-CM98 MM cells (Figure 1). Noteworthy, the molecular weight of NY-ESO-1 expressed by 5-AZA-CdR-treated MPP-89 and MES-CM98 MM cells was identical to that of NY-ESO-1 constitutively expressed by HT1080 fibrosarcoma cells utilised as positive control (Figure 1).

## DISCUSSION

In this study we demonstrate, for the first time, that different immunogenic CTA are concomitantly but heterogeneously expressed in and among human MM specimens. CTA belonging to the MAGE, GAGE and SSX gene families were highly expressed in MM cells analysed (Table 1). This pattern of CTA expression in MM is consistent with the elevated frequency of MAGE family gene expression reported in metastatic melanomas (Brasseur *et al*, 1995); indeed, five out of six MM cells examined expressed MAGE-1 and/or -2, and/or -3 genes (Table 1). Noteworthy, MAGE-3 was highly expressed by MM cells; this finding suggests that MAGE-3, an extensively utilised therapeutic target for cancer immunotherapy (Marchand *et al*, 1999; Nishiyama *et al*, 2001), represents a promising candidate for CTA-based immunotherapy in the majority of MM patients.

Interestingly, the MM cells analysed concomitantly expressed at least four CTA (Table 1), suggesting for the possibility to vaccinate MM patients against multiple therapeutic CTA. Such an approach may serve to reduce the emergence of CTA–negative clones that may escape treatment-induced immune recognition of MM cells. However, the constitutive intratumour heterogeneity of CTA utilised as therapeutic targets (dos Santos *et al*, 2000) could also impair the clinical outcome of CTA-specific immunotherapeutic approaches, through the emergence of CTA-negative neoplastic clones. In this respect, the ability of 5-AZA-CdR to demethylate genomic DNA (Razin and Riggs, 1980), resulting in the induction or up-regulation of different CTA (Table 2, Figure 1), suggests that its *in vivo* administration might revert the CTA-negative phenotype of intratumour MM clones. The feasibility of this approach is further supported by recent evidences indicating that CTA expression induced by 5-AZA-CdR in melanoma cells is long-lasting (Coral *et al*, 1999; De Smet *et al*, 1999), remaining stable throughout cellular replication (De Smet *et al*, 1999).

We also found that 5-AZA-CdR up-regulates the expression of HLA class I antigens, and of the co-stimulatory molecules intercellular adhesion molecule-1 and leukocyte function-associated antigen-3 on MES-MM98 MM cells (data not shown). Thus, *in vivo* administration of 5-AZA-CdR, in addition to induce/up-regulate CTA expression by MM cells, might also enhance their constitutive immunogenicity through the up-regulated expression of distinct components recognised on transformed cells by T lymphocytes.

Due to its suggested immunotherapeutic potential (Gaugler *et al*, 1996; Neumann *et al*, 1998), the distribution of the TAA RAGE-1 was also investigated. Noteworthy, RAGE-1 was expressed in all benign and malignant mesothelial cells investigated, with the exception of MES-CM98 and MES-2 MM cells (Table 1). This pattern of RAGE-1 expression represents a unique feature of mesothelial and mesothelioma cells; in fact, RAGE-1 is rarely expressed in solid malignancies, and only in the retina among normal tissues (Gaugler *et al*, 1996). The distribution of RAGE-1 in benign and malignant mesothelial cells closely resembles that of melanocyte differentiation antigens in melanoma (Traversari, 1999), and suggests for a possible tissue-specificity of RAGE-1 expression.

The results of this study, although preliminary, strongly suggest that CTA-based immunotherapy may represent a suitable therapeutic approach to MM, and provide the scientific background for new and eventually more effective chemo-immunotherapeutic approaches in MM patients.
